# Endogenous enzyme activities and tibia bone development of broiler chickens fed wheat-based diets supplemented with xylanase, β-glucanase and phytase

**DOI:** 10.5713/ajas.19.0885

**Published:** 2020-04-12

**Authors:** Mohammed Al-Qahtani, Emmanuel Uchenna Ahiwe, Medani Eldow Abdallh, Edwin Peter Chang’a, Harriet Gausi, Michael R Bedford, Paul Ade Iji

**Affiliations:** 1 School of Environmental and Rural Sciences, University of New England, Armidale, New South Wales 2351, Australia; 2 Ministry of Education, Riyadh, 12435, Saudi Arabia; 3 AB Vista, 3 Woodstock Court, Marlborough, Wiltshire SN8 4AN, UK; 4 College of Agriculture, Fisheries and Forestry, Fiji National University, P.O. Box 1544, Nausori, Fiji

**Keywords:** Broiler, Enzyme, Tibia, Wheat

## Abstract

**Objective:**

This study assessed the effect of different levels of xylanase, β-glucanase and phytase on intestinal enzyme activities and tibia bone development in broiler chickens fed wheat-based diets.

**Methods:**

Twelve experimental diets were formulated using a 3×2×2 factorial design (three doses of phytase and two doses of both xylanase and β-glucanase) and offered to 648 day-old Ross 308 male chicks having 6 replicates groups with 9 birds per replicate and lasted for 35 days.

**Results:**

An interaction between the enzymes products improved (p<0.01) the activity of chymotrypsin. Protein content at d 10 was highest (p<0.001) with addition of phytase while general proteolytic activity (GPA) (p<0.02) and lipase activity (p<0.001) were decreased. At d 24, there were improvements in protein content (p<0.01) and lipase (p<0.04) with supplementation of superdose phytase. Addition of superdose phytase decreased in chymotrypsin (p<0.02), trypsin (p<0.01) and GPA (p<0.001). The optimum dose of xylanase decreased the chymotrypsin activity (p = 0.05), while the GPA (p<0.001) was increased with the optimum level of β-glucanase. Superdose phytase supplementation at d 10 improved maltase (p = 0.05), sucrase (p<0.001) and alkaline phosphatase (p<0.001) activities in the jejunum while aminopeptidase activity was highest (p<0.005) with the low level of phytase. Protein content of jejunum mucosa was bigger (p<0.001) in birds fed superdose phytase while maltase activity (p<0.001) at d 24 was reduced by this treatment. Sucrase (p<0.04) and aminopeptidase activities (p<0.001) improved when diets supplemented with low levels of phytase. Tibia bone breaking strength was highest (p<0.04) with addition of low level of superdose phytase or optimum level of β-glucanase. Bone dry matter content decreased (p<0.04) when diets supplemented with phytase.

**Conclusion:**

From the results obtained in this study, supplementation of superdose phytase was the most effective, however, the cost-benefit analysis of the use of such a dose needs to be evaluated.

## INTRODUCTION

Wheat is an important energy source in poultry diets in many regions of the world, including Europe, Canada, Australia and New Zealand. However, the nutritive value of wheat is limited by its content of soluble non-starch polysaccharides (NSP), which are indigestible and impede the digestion of other nutrients. Like most grains, wheat also contains phytate, the main reservoir of phosphorus, which is not digested by birds due to their limited secretion of phytase [1]. The exogenous enzymes that were used in this study, xylanase, β-glucanase, and phytase, are now extensively used as additives in poultry diets and their physical effects are generally well understood, but the mechanisms behind their actions are still being researched. Research results to date have been used to determine or predict the optimum dose of xylanase and β-glucanase enzymes in wheat- and barley-based diets and efforts have been made to set the same for phytase in maize-based diets [2]. Although the efficacy of supplemental carbohydrases, proteases, and phytases in diets of poultry has been well established, there is still a great deal of uncertainty on the mechanisms of action of exogenous enzymes. Several factors can influence the response to combinations of enzymes, ranging from enzyme specificity to the target substrate, dosage, interactions between enzymes, ingredient quality, ingredient composition and age of animals. A number of mechanisms have been proposed to explain the useful effects of glucanase in improving energy and nutrient utilization of wheat-based diets [3]. Possible mechanisms of action of carbohydrases in poultry diets include: improved access of endogenous enzymes to cell contents due to hydrolysis of cell wall arabinoxylans [4], reduction in viscosity and provision of prebiotics to stimulate a more beneficial microbiome. Additionally, they have been shown to augment the endogenous digestive enzymes in young animals. In addition to the use of the conventional xylanase, glucanase, phytase, and more recently, multicarbohydrase preparations, the application of normal digestive tract enzymes has also been proposed [5–8]. Several studies on the impact of nutrient restriction on leg abnormalities have concluded that the reduction in leg problems was mostly due to increased activity in birds at a critical stage in leg bone development [9,10]. Phytase improved the concentrations of Fe and Mg in broiler tibia bone but had no effect on Ca, P, and Zn contents [11], however, Shelton and Southern [12] found that the concentration of Zn in tibia was increased while Fe and Mn levels were not affected by dietary phytase. Other studies, [13,14] reported that phytase improved Zn utilization in broilers. Viveros et al [15] also found that phytase supplementation improved Ca, P, Mg and Zn retention in broilers at three and six weeks of age. Żyła et al [16] reported an improvement in gross performance and utilization of energy by chickens when phytase and xylanase were added to wheat-based diets. However, information on the effect of the impact of these enzymes and their combinations on broiler performance, energy utilization and nutrient digestibility is scarce. The present study was aimed at assessing the endogenous enzyme activities and tibia bone development of broiler chickens fed wheat-based diets supplemented with a combination of xylanase, β-glucanase, and phytase.

## MATERIALS AND METHODS

### Experimental design and management of birds

This experiment was designed to investigate the effects of different levels of phytase, xylanase, and β-glucanase supplements in diets fed to chickens between hatch and 10, 24, or 35 days. The enzyme products (Econase XT 25, Econase GT, and Quantum Blue) used were supplied by AB Vista, Marlborough, UK, while the wheat was obtained from local suppliers in New South Wales, Australia. A 3×2×2 factorial study was conducted using three levels of Quantum Blue; none, low (30 mg/kg) and a superdose (300 mg/kg), and none and optimum levels (100 mg/kg) of both Econase XT 25 and Econase GT. The ingredient and nutrient composition of the diets used are shown in Table 1. The basal diets were identical in ingredient profile and formulated to meet the nutrient specifications of broiler chickens as recommended by Aviagen [17]. A total of 648 male day-old Ross 308 broiler chicks (initial weight, 40.45±1.05 g) were randomly assigned to 12 treatments, each with six replicates (9 chickens per replicate). Birds were reared in multi-tiered brooder cages and raised in climate-controlled rooms at the Centre for Animal Research and Teaching, University of New England, Australia. Birds had *ad libitum* access to feed and water over the trial period. The initial brooding temperature was 33°C; this was gradually reduced to 24°C±1°C at 19 days of age and fixed at this level until the end of the experiment. Twenty-four hours of lighting were provided on the first day, then it was reduced to 23 h per day until day 3 after which 18 hours of light were delivered for the remainder of the experiment.

### Growth performance

The leftover feed and birds were weighed at 35 days to measure the body weight gain (BWG), feed intake (FI), and feed conversion ratio (FCR). Feed intake, BWG and FCR data were corrected for mortality.

### Digestive enzyme analyse

At d 10 and d 24 one bird was randomly selected from each cage, weighed, electrically stunned, killed by cervical dislocation and dissected to obtain 1 to 2 cm of the proximal part of the jejunum and the entire pancreas, which were used to measure the endogenous enzyme activities. Both the jejunum and pancreas were wrapped in labelled aluminum foil and snap-frozen in liquid nitrogen until they were transferred to a freezer storage room (−20°C) prior to analysis. The jejunum and pancreatic samples were processed according to the method described by Shirazi-Beechey et al [18] and Nitsan et al [19]. Pancreatic and jejunal enzyme activities were measured by incubation with various substrate concentrations as standardized for poultry [20]. The protein content of tissues and the activities of alkaline phosphatase, maltase, and sucrase were analyzed in the jejunal homogenate. The pancreatic tissue protein content and activities of trypsin and chymotrypsin amidase were assessed as previously described [21,22]. Protein concentrations in pancreatic and jejunal samples were measured by the Coomassie dye-binding procedure [23].

### Apparent metabolizable energy

Excreta was collected into trays underneath each cage between 20 and 23 days. These were pooled per cage and used to determine the apparent metabolizable energy (AME). A sub-sample of excreta was analysed for TiO_2_ and gross energy (GE).

AME was then calculated as: AME = GEi−(GEo×[Ti/To]), where GEi is the gross energy (MJ/kg) in feed, GEo is the gross energy (MJ/kg) in excreta, Ti is the concentration of titanium in the diets, and To is the concentration of titanium in the excreta.

### Titanium dioxide analysis and ileal digestibility of nutrients

At d 24, two birds were randomly selected from each cage, weighed, electrically stunned, euthanazed using cervical dislocation and dissected. The ileal digesta was gently flushed with distilled water into plastic containers. Digesta samples from each cage were pooled together and then freeze-dried, ground (around 0.5 mm pore size) and stored in air-tight containers at −4°C before laboratory analysis. Both digesta and diet samples were analyzed for TiO_2_ according to the method described by Short et al [24].

The apparent ileal nutrient digestibility percentage was calculated by the following formula using TiO_2_ as the indigestible marker:

$$\text{Digestibility }\%=1-\frac{{\text{TiO}}_{2\text{Diet}}\times {\text{N}}_{\text{Digesta}}}{{\text{TiO}}_{2\text{Digesta}}\times {\text{N}}_{\text{Diet}}}\times 100$$

where N_digesta_ is the nutrient concentration in digesta (%), TiO_2Digesta_ is the titanium concentration in digesta (%), N_diet_ is nutrient concentration in diets (%) and TiO_2Diet_ is the titanium dioxide concentration in feeds (%).

### Bone breaking strength

On d 35 the right drumstick was taken from two birds of each replicate and frozen at −20°C for bone strength analyses. The samples were defrosted and the tibia bone was extracted after adherent muscles, tissues, cartilage caps and fibula were removed manually. Breaking strength of the tibia bone was measured by positioning a 10 mm diameter compression rod against the bones and applying pressure (Lloyd, Hampshire, UK). Breaking strength was recorded as the force required to break the bone and was measured in the range of 0 to 500 N. The entire bones were then dried for 12 h at 105°C in a forced-air convection oven (Qualtex Universal Series 2000, Watson Victor Ltd, Perth, Australia) and ashed (550°C for 4 h) in a Carbolite CWF 1200 chamber furnace (Carbolite, Sheffield, UK). The ashed samples were ground and stored at 4°C in airtight plastic containers for dry matter (DM) and mineral content analyses.

### Animal ethics

The Animal Ethics Committee of the University of New England, Australia approved the study (approval number AEC15-080).

### Statistical analyses

The data were statistically analysed using the general linear model procedure of Minitab version 17 software program [25]. Tukey’s test was used to compare mean values for significant differences at the p≤5% level of probability.

## RESULTS

### Gross response

Results of FI, BWG, and FCR between one and 35 days of age are shown in Table 2. There was an interaction (p<0.03) between phytase and β-glucanase, which improved the FCR from hatch to 35 d. Feed intake was decreased (p<0.001) with supplementation of optimum level of xylanase. From one to 35 d, birds fed the low dose of phytase had higher BWG compared to the other groups. Supplementing diets with optimum dose of xylanase improved (p = 0.05) BWG during 1 to 35 d. The addition of optimum level of xylanase between one and 35 d resulted in a better (p<0.001) FCR than the control.

### Endogenous enzyme activities

Chymotrypsin activity at d 10 was improved (p<0.01) due to an interaction between the three enzyme products. Protein content at d 10 improved (p<0.001) with addition of phytase while general proteolytic activity (GPA) (p<0.02) and lipase activity (p<0.001) decreased with addition of phytase (Table 3). The pancreatic protein content and enzyme activities at d 24 are shown in Table 4. There were increments in protein content (p<0.01) and lipase activity (p<0.04) with supplementation of superdose level of phytase. On the other hand, increasing the supplemented led phytase resulted in a decrease in chymotrypsin (p<0.02), trypsin (p<0.01), and GPA (p<0.001). The optimum dose of xylanase also decreased the chymotrypsin activity (p = 0.05), while optimum level of β-glucanase increased the GPA (p<0.001).

Jejunal enzyme activities at 10 and 24 d of age are presented in Table 5 and 6, respectively. Phytase superdose improved maltase (p = 0.05), sucrase (p<0.001), and alkaline phosphatase (p<0.001) activities at 10 d of age while the low level of phytase addition increased aminopeptidase activity (p<0.005). At d 24, protein content (p<0.001) of the jejunual mucosa and activity of sucrase (p<0.04) were increased by phytase supplementation while maltase activity (p<0.001) was reduced. Aminopeptidase activity peaked (p<0.001) at the low level of phytase. No effects of interactions were observed.

### Apparent metabolizable energy and nutrient digestibility

An interaction (p<0.01) between phytase, xylanase and β-glucanase resulted to an improvement in gross energy digestibility. There was an interaction (p<0.01) between phytase and β-glucanase, which resulted in increased starch digestibility. Apparent metabolizable energy increased (p<0.01) when diets were augmented with each of the enzymes, irrespective of the dosage administered (Table 7). There was an improvement in crude protein digestibility with addition of phytase (p<0.001) and optimum level of β-glucanase (p< 0.003), but this was not significant (p>0.05) with xylanase.

### Breaking strength and mineral contents of tibia bone

Table 8 shows the effects of the test enzymes on bone strength. Tibia bone breaking strength increased with addition of doses of phytase (p<0.001) and at optimum level of β-glucanase (p<0.04). Bone DM content decreased (p<0.04) when diets were supplemented with phytase. There was no significant in mineral contents of tibia bone.

## DISCUSSION

### Gross response

The results showed that the gross performance of broiler chickens was affected by the test enzyme supplements between one and 35 days. This observation could be attributed to the combined action of phytase on phytic acid and xylanase on the xylans as well as the breakdown of glucans by β-glucanase. These enzyme interactions help to increase the digestibility of nutrients in young birds as they lack these enzymes, with a resultant increase in feed conversion. This result has been demonstrated by Peng et al [26] who reported an increase in FI by xylanase supplementation during 1 to 3 weeks of age but the effect was reduced during 4 to 6 weeks. It is well known that phytate negatively affects protein availability and absorption of some minerals [27], and increases mucus production [28] thereby, reducing broiler performance. The use of phytase in broiler diets to degrade phytate and thus release phosphorus and certain other nutrients and improve productivity is a common practice in commercial broiler production [29]. The current results confirm the benefit of feeding a superdose level of phytase as the performance is significantly better than that of those birds fed the low or more conventional levels of phytase.

### Endogenous enzyme activities

Phytase supplementation increased the pancreatic protein content and chymotrypsin activity but reduced lipase activity at d 10 while at d 24 this observation changed to a reduction in chymotrypsin activity, an increase in lipase, GPA and total protein content especially with the superdose level of phytase. Increased levels of chymotrypsin at d 10 of age may be reflecting improved gastric digestion due to the reduction of phytate inhibition, enabling greater through-flow of protein and hence a greater demand for pancreatic enzymes. At 24 d of age this effect was reversed, possibly because a larger pancreas is more able to cope and the need for chymotrypsin is no longer the bottleneck when gastric and small intestinal proteolysis is eased with superdose phytase. Fuente et al [30] reported that viscosity of the intestinal contents determined with 30-d-old chickens was negatively related to endogenous β-glucanase activity. The inverse effects on lipase is interesting and may reflect differential changes in the lipolytic capacity of the bird.

Jejunal enzyme activities seem generally to increase with high phytase supplementation in particular at d 10 but this effect is largely lost at d 24 with the exception of sucrase activity. According to Pinheiro et al [31] a complex enzymes supplementation had an through an interaction effect resulted in improved enzyme activity and nutrient digestibility immediately after a feed restriction period from 7 to 14 d. There is marked effects of phytase superdosing on jejunal alkaline phosphatase at 10 d. This enzyme plays a critical role in intestinal integrity and reduction of inflammatory responses. Further, it is thought to dephosphorylate myo-inositol monophosphate (IP_1_) [32], the end product of phytase activity, which might explain why it is so responsive to phytase superdosing. If IP_1_, as a substrate, induces alkaline phosphatase activity then this may partly explain the performance benefits noted with superdosing phytase. Reports of studies on endogenous enzyme activities in broilers fed wheat-based diets supplemented with phytase are limited. However, the change may be a reflection of an impact on endogenous enzymes. The test enzymes tended to accentuate rather than reduce the activities of the endogenous enzymes. It is difficult to compare the present study to previous studies because this study used different doses of carbohydrases and phytase and there is a need to do more research in this area.

### Apparent metabolizable energy and nutrient digestibility

Apparent metabolizable energy improved with supplementation of phytase, xylanase, and β-glucanase. The action of these enzymes on anti-nutrients such as NSP, phytic acid, and other factors, although not directly measured, might be the main reason behind the improvement in the AME and the fact that these were independent main effects suggests they are additive and possibly working through different mechanisms. This result agree with the finding of Wu et al [33] who observed that the use of phytase and xylanase increased the AME and digestibility of nutrients in wheat-based diets for broilers. β-Glucanase was also shown to improve the AME in this study. It is noteworthy that the effect of phytase was considerably larger than that of either the xylanase or β-glucanase alone. Bedford [2] reported that large influxes of digestive enzymes, bile acids, lecithin and lysozyme are a challenge to gut microbes such that the duodenum is largely devoid of bacteria.

Phytase addition improved protein digestibility, as has been previously reported [2,34] who indicated that adding phytase to broiler diets improves the digestibility of protein and amino acids. The positive effect of xylanase on protein digestibility has also been reported [33]. Individual effects of xylanase on starch digestibility and all three enzymes on gross energy were observed in this study. Ravindran et al [35] reported that energy digestibility was improved by dietary phytase supplementation, while Liu et al [36] demonstrated that adding xylanase to wheat-based diets reduced the intestinal mucosal viscosity and improved the digestibility of energy and starch in broiler chickens. β-Glucanase has been observed to improve energy and starch digestibility when supplemented alone or combined with xylanase and/or phytase to wheat and barley-based diets [33,37,38].

In the current study, more than two interactions were noticed between the three test enzymes on digestibility of arginine, threonine, leucine, and lysine. Phytase and β-glucanase supplementation increased the digestibility of almost all the measured amino acids but the benefit of the phytase was markedly greater. These results are in line with the improvement in protein digestibility, which was previously highlighted. It has been shown that phytate-protein bonds are insoluble and less responsive to proteolytic enzymes than protein alone [39]. This binding could reduce the solubility and therefore, digestibility of proteins and amino acids.

### Bone breaking strength and mineral content of tibia bone

Tibia bone breaking strength was improved with the addition of phytase or β-glucanase. The test enzymes had no effect on the mineral contents of the bones in the present study, suggesting that the beneficial effect on breaking strength may be due to change in bone matrix rather than mineralisation. It has been reported that adding phytase to broiler chicken diets results in a marked improvement in the utilization of phytate phosphorus as measured by bone ash, and bone strength [40]. Bone density is considered to reflect bone mineral content. However, many recent studies have shown that the mineral density can be contingent upon the chemical organic matrix of bones [41].

## CONCLUSION

The test microbial enzymes (phytase, xylanase, and β-glucanase) in wheat-based diets especially at superdose level in the case of phytase, improved the utilization of several enzyme activities that was assessed in the present study. Furthermore, supplementation of phytase, xylanase, and β-glucanase improved gross performance, possibly through increased nutrient digestibility as well as improved breaking strength of the tibia bone. The inclusion of a superdose level of phytase has even more benefits, although the economics of use of such a dose has not been evaluated.

## Figures and Tables

**Table 1:** Ingredient and nutrient composition of diets fed

Items	Starter1)	Grower1)	Finisher1)
Ingredient composition (%)			
Wheat	60.45	64.13	68.28
Soybean meal	29.41	22.78	19.53
Meat and bone meal	3.00	5.00	4.00
Canola oil	3.58	4.50	5.49
Limestone	0.94	0.66	0.73
Dicalcium phosphate	1.16	0.54	0.70
Salt	0.11	0.08	0.10
Na bicarb	0.20	0.20	0.20
TiO2	0.00	0.50	0.00
Vit-mineral premix2)	0.20	0.20	0.20
Choline Cl 70%	0.05	0.05	0.05
L-lysine	0.33	0.86	0.29
DL-methionine	0.36	0.32	0.28
L-threonine	0.21	0.18	0.15
Total	100	100	100
Nutrient composition (%)			
ME (MJ/kg)	12.55	12.97	13.40
Crude protein	23.00	21.50	19.50
Crude fat	5.51	6.55	7.47
Crude fibre	2.49	2.41	2.37
Arginine	1.37	1.23	1.09
Lysine	1.28	1.17	1.02
Methionine	0.65	0.58	0.52
Methionine+cysteine	0.95	0.87	0.80
Tryptophan	0.26	0.23	0.21
Isoleucine	0.91	0.82	0.75
Threonine	0.86	0.77	0.68
Valine	1.01	0.93	0.85
Calcium	0.96	0.87	0.84
Available P	0.48	0.43	0.39
Sodium	0.16	0.16	0.16
Potassium	0.94	0.82	0.74
Chlorine	0.21	0.31	0.20
Choline (mg/kg)	1,700	1,600	1,500
Linoleic	1.71	1.92	2.17

ME, metabolizable energy.

1) Starter, grower, and finisher were each one are 12 treatments 3 levels of phytase, 2 levels of both xylanase and β-glucanase.

2) The active ingredients contained in the vitamin–mineral premix were as follows (per kg of diet): vitamin A 12,000 IU, vitamin D_3_ 3,500 IU, vitamin E 30.0 mg, vitamin K_3_ 2.0 mg, thiamine 2 mg, riboflavin 6 mg, pyridoxine 5 mg, vitamin B_12_ 0.02 mg, niacin 50 mg, pantothenate 12 mg, biotin 0Æ01 mg, folic acid 2 mg, Fe 60 mg, Zn 60 mg, Mn 80 mg, Cu 8 mg, Se 0Æ1 mg, Mo 1 mg, Co 0Æ3 mg, I 1 mg.

**Table 2:** Gross response of birds on diets containing different levels of phytase, xylanase, and β-glucanase fed between hatch and 35 d of age

Enzymes levels			FI (kg/bird)	BWG (kg/b)	FCR
Phytase	**Xylanase**	**β-glucanase**	**1–35 d**	**1–35 d**	**1–35 d**
None	None	None	3.56	2.37	1.50^a^
	Optimum	None	3.33	2.41	1.38abc
	None	Optimum	3.57	2.43	1.47^ab^
	Optimum	Optimum	3.36	2.43	1.39abc
Low	None	None	3.62	2.47	1.46^ab^
	Optimum	None	3.54	2.51	1.41abc
	None	Optimum	3.62	2.48	1.46^ab^
	Optimum	Optimum	3.45	2.59	1.33^c^
Superdose	None	None	3.55	2.51	1.42abc
	Optimum	None	3.34	2.53	1.32^c^
	None	Optimum	3.54	2.39	1.48^ab^
	Optimum	Optimum	3.46	2.53	1.37^bc^
SEM			24.60	15.20	0.009
Main effects					
None			3.46	2.41^b^	1.43
Low			3.56	2.51^a^	1.42
Superdose			3.47	2.49^ab^	1.40
	None		3.58^a^	2.44^b^	1.46^a^
	Optimum		3.41^b^	2.50^a^	1.37^b^
		None	3.49	2.47	1.42
		Optimum	3.50	2.47	1.42
Source of variation					
Phytase			0.17	0.02	0.13
Xylanase			0.001	0.05	0.001
β-glucanase			0.85	0.82	0.98
Phytase × xylanase			0.71	0.62	0.93
Phytase × β-glucanase			0.64	0.29	0.03
Xylanase × β-glucanase			0.84	0.38	0.53
Phytase × xylanase × β-glucanase			0.65	0.46	0.27

Values are means of 6 replicates (9 birds from each cage).

FI, feed intake; BWG, body weight gain; FCR, feed conversion ratio (kg feed:kg body weight gain); SEM, standard error of means.

a–c Mean values with different superscripts within the columns are different (p<0.05).

**Table 3:** Effect of diets containing different levels of phytase, xylanase, and β-glucanase on pancreatic protein concentration (mg/g tissue) and enzyme activities (μmol/mg protein/min) (10 days of age)

Phytase levels	Xylanase levels	β-glucanase levels	Protein	ChymoT	Trypsin	GPA	Lipase
None	None	None	88.3	3.10^b^	3.80	0.75	7.20
	Optimum	None	84.2	4.00^ab^	4.00	0.77	7.50
	None	Optimum	80.0	4.30^a^	4.20	0.87	7.70
	Optimum	Optimum	88.4	2.80^b^	4.00	0.70	6.80
Low	None	None	107.5	4.00^ab^	3.80	0.70	6.30
	Optimum	None	107.0	3.30^ab^	3.10	0.68	5.90
	None	Optimum	108.4	3.20^ab^	3.20	0.70	6.20
	Optimum	Optimum	106.1	4.30^a^	3.60	0.70	6.80
Superdose	None	None	104.7	4.70^a^	4.20	0.77	5.30
	Optimum	None	99.3	4.30^a^	4.10	0.74	5.50
	None	Optimum	109.8	4.20^a^	4.10	0.74	5.80
	Optimum	Optimum	114.7	4.70^a^	3.6	0.75	5.40
SEM			1.93	0.14	0.12	0.01	0.13
Main effects							
None			85.2^b^	3.50^b^	4.00	0.77^a^	7.30^a^
Low			107.3^a^	3.70^ab^	3.50	0.70^b^	6.30^b^
Superdose			107.1^a^	4.40^a^	4.00	0.75^ab^	5.50^c^
	None		99.8	3.90	3.90	0.75	6.40
	Optimum		100.0	3.90	3.80	0.73	6.30
		None	98.5	3.90	3.80	0.74	6.30
		Optimum	101.3	3.90	3.80	0.74	6.50
Source of variation							
Phytase			0.001	0.02	0.10	0.02	0.001
Xylanase			0.96	0.95	0.56	0.19	0.59
β-Glucanase			0.38	0.92	0.87	0.72	0.38
Phytase × xylanase			0.89	0.71	0.95	0.30	0.72
Phytase × β-glucanase			0.22	0.97	0.65	0.83	0.44
Xylanase × β-glucanase			0.26	0.85	0.86	0.34	0.41
Phytase × xylanase × β-glucanase			0.59	0.01	0.37	0.08	0.06

Values are means of 6 replicates (one bird per cage).

ChymoT, chymotrypsin; GPA, general proteolytic activity; SEM, standard error of means.

a–c Mean values with different superscripts within the columns are different (p<0.05).

**Table 4:** Effect of diets containing different levels of phytase, xylanase, and β-glucanase on pancreatic protein concentration (mg/g tissue) and enzyme activities (μmol/mg protein/min) (24 days of age)

Phytase levels	Xylanase levels	β-glucanase levels	Protein	ChymoT	Trypsin	GPA	Lipase
None	None	None	122.5	3.20	3.10	0.52	4.40
	Optimum	None	99.9	2.70	3.10	0.54	4.20
	None	Optimum	94.1	3.10	2.80	0.60	4.30
	Optimum	Optimum	101.8	2.40	2.40	0.60	4.30
Low	None	None	112.1	2.30	2.20	0.50	4.20
	Optimum	None	105.2	2.60	2.70	0.50	4.10
	None	Optimum	109.1	3.20	2.90	0.57	4.40
	Optimum	Optimum	111.1	2.40	2.20	0.56	4.50
Superdose	None	None	121.7	2.40	2.40	0.50	5.20
	Optimum	None	135.3	2.20	2.20	0.48	4.50
	None	Optimum	132.5	2.30	2.50	0.49	5.10
	Optimum	Optimum	126.1	2.10	2.30	0.51	4.50
SEM			2.68	0.09	0.08	0.08	0.09
Main effects							
None			104.6^b^	2.90^a^	2.90^a^	0.56^a^	4.30^b^
Low			109.4^b^	2.60^ab^	2.50^ab^	0.53^ab^	4.30^b^
Superdose			128.9^a^	2.30^b^	2.40^b^	0.49^b^	4.80^a^
	None		115.3	2.80^a^	2.70	0.53	4.60
	Optimum		113.2	2.40^b^	2.50	0.53	4.40
		None	116.1	2.60	2.60	0.51^b^	4.40
		Optimum	112.5	2.60	2.5	0.55^a^	4.50
Source of variation							
Phytase			0.001	0.02	0.01	0.001	0.04
Xylanase			0.67	0.05	0.28	0.81	0.19
β-Glucanase			0.45	0.84	0.52	0.001	0.69
Phytase × xylanase			0.65	0.64	0.97	0.89	0.47
Phytase × β-glucanase			0.38	0.42	0.14	0.22	0.70
Xylanase × β-glucanase			0.51	0.26	0.10	0.93	0.64
Phytase × xylanase × β-glucanase			0.11	0.34	0.19	0.58	1.00

Values are means of 6 replicates (one bird per cage).

ChymoT, chymotrypsin; GPA, general proteolytic activity; SEM, standard error of means.

a,b Mean values with different superscripts within the columns are different (p<0.05).

**Table 5:** Effect of diets containing different levels of phytase, xylanase, and β-glucanase on jejunual protein concentration (mg/g tissue) and enzyme activities (μmol/mg protein/min) in jejunum (10 days of age)

Phytase levels	Xylanase levels	β-glucanase levels	Protein	Maltase	Sucrase	AP	APP
None	None	None	53.9	0.68	0.06	0.56	4.10
	Optimum	None	56.0	0.72	0.07	0.59	3.40
	None	Optimum	53.8	0.66	0.06	0.56	3.60
	Optimum	Optimum	53.4	0.79	0.07	0.59	4.00
Low	None	None	53.7	0.79	0.09	0.70	4.80
	Optimum	None	51.7	0.80	0.11	0.73	4.50
	None	Optimum	60.5	0.71	0.08	0.57	3.80
	Optimum	Optimum	54.8	0.86	0.10	0.80	4.60
Superdose	None	None	58.9	0.82	0.10	0.74	3.70
	Optimum	None	55.8	0.80	0.11	0.86	3.80
	None	Optimum	55.6	0.85	0.13	0.89	3.70
	Optimum	Optimum	57.2	0.79	0.10	0.79	3.70
SEM			1.21	0.02	0.004	0.03	0.10
Main effects							
None			54.3	0.72^b^	0.07^c^	0.58^b^	3.80^b^
Low			55.2	0.79^b^	0.09^b^	0.70^ab^	4.40^a^
Superdose			56.9	0.82^a^	0.11^a^	0.82^a^	3.70^b^
	None		56.1	0.75	0.09	0.67	4.00
	Optimum		54.8	0.79	0.09	0.73	4.00
		None	55.0	0.77	0.90	0.70	4.00
		Optimum	55.9	0.78	0.90	0.70	3.9
Source of variation							
Phytase			0.70	0.05	0.001	0.001	0.005
Xylanase			0.63	0.26	0.23	0.27	0.92
β-Glucanase			0.73	0.82	0.93	0.93	0.46
Phytase × xylanase			0.74	0.24	0.10	0.54	0.79
Phytase × β-glucanase			0.53	0.90	0.28	0.86	0.51
Xylanase × β-glucanase			0.92	0.37	0.15	0.97	0.08
Phytase × xylanase × β-glucanase			0.77	0.51	0.26	0.23	0.35

Values are means of 6 replicates (one bird per cage).

AP, alkaline phosphatase; APP, aminopeptidase; SEM, standard error of means.

a–c Mean values with different superscripts within the columns are different (p<0.05).

**Table 6:** Effect of diets containing different levels of phytase, xylanase, and β-glucanase on jejunual protein concentration (mg/g tissue) and enzyme activities (μmol/mg protein/min) in jejunum (24 days of age)

Phytase levels	Xylanase levels	β-Glucanase levels	Protein	Maltase	Sucrase	AP	APP
None	None	None	83.3	0.56	0.06	0.26	1.25
	Optimum	None	93.2	0.61	0.05	0.19	1.13
	None	Optimum	89.2	0.65	0.05	0.25	1.24
	Optimum	Optimum	83.7	0.58	0.06	0.26	1.19
Low	None	None	87.5	0.59	0.06	0.25	1.44
	Optimum	None	84.6	0.53	0.06	0.29	1.27
	None	Optimum	88.2	0.60	0.06	0.25	1.36
	Optimum	Optimum	81.7	0.60	0.06	0.29	1.39
Superdose	None	None	111.5	0.50	0.06	0.21	1.10
	Optimum	None	113.3	0.48	0.06	0.22	1.02
	None	Optimum	122.6	0.46	0.06	0.24	1.03
	Optimum	Optimum	111.4	0.48	0.06	0.25	1.11
SEM			2.97	0.01	0.001	0.01	0.03
Main effects							
None			87.4^b^	0.60^a^	0.05^b^	0.24	1.20^b^
Low			85.5^b^	0.58^a^	0.06^a^	0.27	1.37^a^
Superdose			114.7^a^	0.48^b^	0.06^ab^	0.23	1.06^b^
	None		97.0	0.56	0.06	0.24	1.24
	Optimum		94.6	0.55	0.06	0.25	1.18
		None	95.6	0.54	0.06	0.24	1.20
		Optimum	96.1	0.56	0.06	0.26	1.22
Source of variation							
Phytase			0.001	0.001	0.04	0.38	0.001
Xylanase			0.66	0.53	0.67	0.83	0.29
β-Glucanase			0.92	0.42	0.86	0.37	0.68
Phytase × xylanase			0.83	0.83	0.98	0.49	0.77
Phytase × β-glucanase			0.87	0.54	0.87	0.86	0.99
Xylanase × β-glucanase			0.33	0.73	0.30	0.67	0.16
Phytase × xylanase × β-glucanase			0.90	0.20	0.56	0.75	0.88

Values are means of 6 replicates (one bird per cage).

AP, alkaline phosphatase; APP, aminopeptidase; SEM, standard error of means.

a,b Mean values with different superscripts within the columns are different (p<0.05).

**Table 7:** Apparent metabolizable energy and ileal nutrient digestibility of birds on diets supplemented with different levels of phytase, xylanase, and β-glucanase (24 days of age)

Phytase levels	Xylanase levels	β-Glucanase levels	AME	Protein	GE	Starch
None	None	None	13.51	81.07	76.54^d^	95.75^ab^
	Optimum	None	13.73	80.09	75.36^d^	94.95^ab^
	None	Optimum	13.96	82.70	77.07^d^	95.26^ab^
	Optimum	Optimum	14.00	82.14	81.19^bc^	95.97^ab^
Low	None	None	14.10	82.60	76.49^d^	94.24^b^
	Optimum	None	14.14	84.83	81.25^bc^	95.07^ab^
	None	Optimum	14.26	86.04	82.67abc	96.69^a^
	Optimum	Optimum	14.44	85.21	81.68abc	96.34^ab^
Superdose	None	None	14.96	86.37	81.11^c^	96.93^a^
	Optimum	None	15.02	88.24	85.79^a^	96.93^a^
	None	Optimum	15.06	87.92	84.48^ab^	96.89^a^
	Optimum	Optimum	15.40	87.69	84.89^a^	96.79^a^
SEM			0.07	0.40	1.00	1.00
Main effects						
None			13.80^c^	81.50^c^	77.54^c^	95.48^b^
Low			14.23^b^	84.67^b^	80.52^b^	95.59^b^
Superdose			15.11^a^	87.55^a^	83.79^a^	96.89^a^
	None		14.30^b^	84.45	79.73^b^	95.96
	Optimum		14.46^a^	84.70	81.51^a^	96.01
		None	14.25^b^	83.87^b^	79.24^b^	95.64^b^
		Optimum	14.51^a^	85.28^a^	82.00^a^	96.32^a^
Source of variation						
Phytase			0.001	0.001	0.001	0.001
Xylanase			0.01	0.58	0.001	0.86
β-Glucanase			0.001	0.003	0.001	0.01
Phytase × xylanase			0.70	0.29	0.85	0.87
Phytase × β-glucanase			0.58	0.36	0.23	0.01
Xylanase × β-glucanase			0.57	0.09	0.13	0.89
Phytase × xylanase × β-glucanase			0.33	0.28	0.001	0.10

Values are means of 6 replicates (2 birds from each cage) and for AME (8 birds from each cage).

AME, apparent metabolizable energy; GE, gross energy; SEM, standard error of means.

a–d Mean values with different superscripts within the columns are different (p<0.05).

**Table 8:** Breaking strength and mineral contents of tibia bone on 35-day old chicks fed wheat-based diets supplemented with phytase, xylanase, and β-glucanase

Phytase levels	Xylanase levels	β-glucanase levels	BreakStr (kg/mm^2^)	DM (%)	Ash (%)	Ca (%)	P (%)	Mg (%)	K (%)	S (%)
None	None	None	330.0	69.6	49.7	37.2	16.5	0.81	0.59	0.24
	Optimum	None	361.0	68.8	49.4	37.6	16.6	0.82	0.59	0.23
	None	Optimum	361.0	70.0	49.5	37.2	16.5	0.80	0.61	0.24
	Optimum	Optimum	391.5	68.9	49.8	37.1	16.4	0.81	0.57	0.26
Low	None	None	377.2	67.8	49.5	37.4	16.6	0.84	0.56	0.26
	Optimum	None	402.3	66.1	50.0	37.5	16.6	0.83	0.60	0.24
	None	Optimum	405.3	67.2	49.4	37.1	16.4	0.81	0.55	0.25
	Optimum	Optimum	408.8	66.5	49.6	36.9	16.4	0.80	0.59	0.25
Superdose	None	None	396.9	68.7	49.4	36.8	16.1	0.81	0.57	0.26
	Optimum	None	405.3	66.1	49.7	37.2	16.5	0.82	0.60	0.27
	None	Optimum	406.9	66.9	50.1	37.2	16.6	0.83	0.61	0.24
	Optimum	Optimum	412.7	65.6	49.9	36.7	16.3	0.82	0.61	0.26
SEM			5.06	0.44	0.08	0.11	0.05	0.003	0.01	0.004
Main effects										
None			360.9^b^	69.3^a^	49.6	37.3	16.5	0.81	0.59	0.24
Low			398.4^a^	66.9^b^	49.6	37.2	16.5	0.82	0.58	0.25
Superdose			405.6^a^	66.8^b^	49.8	37.0	16.4	0.82	0.60	0.26
	None		379.6	68.4	49.6	37.1	16.5	0.82	0.58	0.25
	Optimum		397.0	67.0	49.7	37.2	16.5	0.82	0.59	0.25
		None	378.9^b^	67.8	49.6	37.3	16.5	0.82	0.58	0.25
		Optimum	397.7^a^	67.5	49.7	37.0	16.4	0.81	0.59	0.25
Source of variation										
Phytase			0.001	0.04	0.57	0.54	0.72	0.41	0.58	0.41
Xylanase			0.06	0.13	0.44	0.94	0.98	0.91	0.43	0.48
β-Glucanase			0.04	0.70	0.48	0.30	0.73	0.09	0.74	0.89
Phytase × xylanase			0.56	0.89	0.51	0.93	0.99	0.43	0.27	0.52
Phytase × β-glucanase			0.80	0.80	0.24	0.71	0.41	0.15	0.63	0.32
Xylanase × β-glucanase			0.64	0.71	0.98	0.21	0.21	0.49	0.59	0.22
Phytase × xylanase × β-glucanase			0.87	0.93	0.35	0.83	0.42	0.76	0.88	0.99

Values are means of 6 replicates (2 birds per cage).

BreakStr, Breaking strength; DM, dry matter; SEM, standard error of means.

a,b Mean values with different superscripts within the columns are different (p<0.05).
